# Multiple Cardiac Diseases Involving the Aortic Arch: Beating Heart Debranching, and Normothermic Arch Replacement: A Case Series

**DOI:** 10.3390/jcm13030732

**Published:** 2024-01-26

**Authors:** Alessandro Motta, Cristian Scarpari, Ermelinda Borrelli, Francesco Formica

**Affiliations:** 1 UO Cardiochirurgia, APSS—Ospedale Santa Chiara, Largo Medaglie d’Oro, 38100 Trento, Italy; cristian.scarpari@apss.tn.it (C.S.); lindaborrelli91@gmail.com (E.B.); 2 UO Cardiochirurgia, Salus Hospital, 42123 Reggio Emilia, Italy; 3 Department of Medicine and Surgery, University of Parma, 43126 Parma, Italy

**Keywords:** aortic arch replacement, frozen elephant trunk, debranching, cerebral perfusion

## Abstract

(1) **Background**: Conventional open surgery is still the gold standard for aortic arch disease, and despite recent developments in optimizing strategies for neuroprotection, distal organ perfusion, and myocardial protection, aortic arch replacement is still associated with high morbidity and mortality rates. (2) **Methods**: We present our case series of 12 patients undergoing surgical management of multiple cardiac diseases involving the aortic arch. In this single-center study, we report our initial experience over a five-year period (from December 2018 to October 2023) with the use of a “debranching first” technique for the supra-aortic vessels of a beating heart, followed by the cardiac step addressing proximal diseases, and a final distal step treating the aortic arch. This strategy aims to minimize cardiac, cerebral, and peripheral ischemia. (3) **Results**: Six patients underwent aortic root replacement with either Bentall (*n* = 4) or valve-sparing aortic root (David procedure) (*n* = 2). The mean nasopharyngeal temperature was 34 °C and the mean cardiocirculatory arrest was 14.3 min. The early mortality was 8.3% (1 patient); no patient experienced a permanent neurologic event. (4) **Conclusions**: In patients with complex aortic disease and concomitant cardiac disease, this approach reduces the need for hypothermia and decreases cardiopulmonary bypass time and myocardial arrest time and therefore could represent a valid surgical option, even in high-risk patients.

## 1. Introduction

The technical approach to complex aortic disease involving the aortic arch remains cumbersome, although it has dramatically evolved over the last decade. It is known that aortic arch replacement in complex aortic diseases, such as type A aortic dissection (TAAD), intramural hematoma (IMH), penetrating aortic ulcer (PAU), and aortic arch aneurysms, is a technically challenging surgical procedure which is associated with a high rate of peri- and postoperative complications and early mortality. Hypothermic circulatory arrest, prolonged cardiopulmonary bypass (CPB) and myocardial arrest times, and prolonged cooling and rewarming periods may result in impaired cerebral, myocardial, and distal organ perfusion and coagulopathy disorders, resulting in increased postoperative morbidity and mortality. The “branch-first” concept was proposed by Nishimura et al. in 2002 [1] and further developed by Matalanis et al. [2,3]. Moreover, the results of a technique combining branch-first and “frozen elephant trunk” (FET) were reported with substantial differences even between different countries [4]. Overall, the main purpose of this approach is to reduce the myocardial ischemic time, to minimize the circulatory arrest time, and to avoid deep and even mild hypothermic core temperature.

Branch-first and FET techniques allow us to manage aortic arch disease with almost continuous cerebral perfusion and a short period of body circulatory arrest with only mild hypothermia. The issue of myocardial protection in those operations involving isolated aortic root disease or concomitant mitral or coronary artery disease needs close attention. In these patients, aortic cross clamping time is often very long and can increase the risk of various grades of myocardial damage despite using appropriate protection techniques.

Although the outcomes of using branch-first techniques alone or in addition to FET appear to be promising, this approach remains challenging, and the results reported from high-volume aortic centers cannot be generalized. Therefore, we describe our initial experience with the surgical approach combining branch-first, FET, and normothermia in patients with complex aortic disease involving the aortic arch together with aortic root or other associated cardiac diseases.

## 2. Materials and Methods

### 2.1. Patient Population

This study represents a case series retrospective study. From December 2018 to October 2023, 12 patients who underwent “complex” operations involving aortic arch replacement in our institution were enrolled. The criteria for patient selection were based on the anatomy and disease of the aortic arch, the concomitant cardiac disease, and the timing of surgery. Complex aortic diseases, such as TAAD, IMH, PAU, aortic arch aneurysms and malperfusion syndrome, were defined according to the most recent guidelines [5].

### 2.2. Surgical Technique

**Figure 1:**
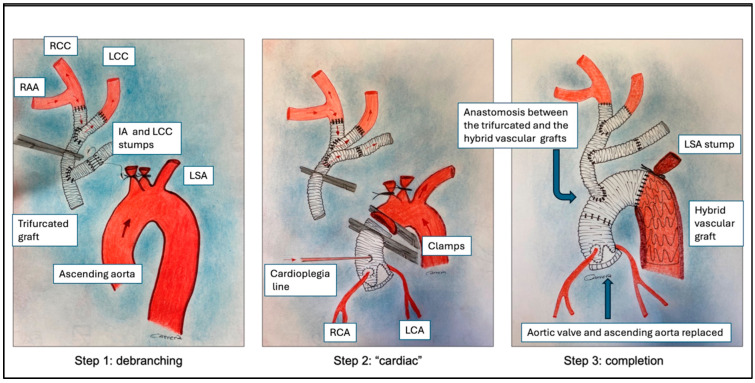
Operative technique steps. RAA, right axillary artery; RCC, right carotid artery; LCC, left carotid artery; IA, innominate artery; LSA, left subclavian artery; LCA, left coronary artery; RCA, right coronary artery.

The operative technique was the same in all cases, as described in Figure 1.

Intraoperative cerebral monitoring was monitored by a combination of cerebral oxygen saturation (near-infrared reflectance spectroscopy—NIRS) and bispectral index (BIS) monitoring. Both radial arteries were cannulated for continuous arterial blood pressure monitoring. Body temperature was monitored by pharyngeal and bladder probes.

Following the induction of anesthesia, the right axillary artery (RAA) was exposed and, after full heparinization, an interposition 8 mm vascular graft was anastomosed to the RAA (termino-lateral continuous 5-0 polypropylene running suture) to insert the return arterial cannula; the right femoral artery (RFA) was also exposed and cannulated directly. Both arterial cannulas were connected to the arterial arm of the CPB circuit with a “Y” connector. After a full median sternotomy, the left axillary artery (LAA) was exposed, and an 8 mm graft was anastomosed and tunneled in the anterior mediastinum. A 12-8-8 mm trifurcated conduit was prepared beforehand (size was based on computed tomography scan measurements). The right atrium was cannulated to initiate the CPB. The innominate artery (IA) and the left carotid artery (LCA) were mobilized. Under partial CPB via RAA (up to approximately 25% total indexed blood flow) and applied to a beating heart, IA and LCA were then sequentially clamped and sewn, respectively, to the 12 mm and 8 mm branches of the trifurcated vascular graft, maintaining continuous cerebral perfusion. LCA perfusion was interrupted for a few minutes during anastomosis with an 8 mm branch. The two stumps were ligated on the aortic arch side. The third branch (8 mm) of the trifurcated graft was then sutured to the remaining 8 mm side graft of LAA (Figure 1, Step 1). Therefore, the clamp was moved to occlude the 12 mm graft proximally to the trifurcation in order to ensure the perfusion of the brain and the upper body through the RAA (Figure 1, Step 2). Total CBP was started via RAA and RFA, leaving the temperature to drop at about 34 °C spontaneously. The ascending aorta was clamped, and the heart was arrested by the administration of intermittent antegrade and retrograde cold blood cardioplegia. The aortic root and coronary artery disease and/or mitral valve disease were addressed where necessary. The proximal aortic graft was then distally clamped, and heart perfusion was established through a modified cardioplegia line inserted into the vascular graft (Figure 1, Step 2).

Systemic circulatory arrest was established by clamping the femoral artery and adapting the blood flow on RSA. The native arch was removed in zone 2, the left subclavian artery origin was ligated, and an E-Vita Open 33/130 prosthesis (Jotec Gmbh, Hechingen, Germany) was advanced and deployed in the descending thoracic aorta. An armed silicon Foley catheter was then introduced through the side port of the E-Vita prosthesis and inflated into the E-Vita aortic thoracic segment to occlude the distal aorta and to re-establish the distal perfusion through the femoral artery. The distal anastomosis of the E-Vita and the aortic stump and the anastomosis between the aortic graft and E-Vita graft were performed sequentially. The Foley catheter was removed, and systemic circulation was re-established via the femoral artery. The proximal end of the trifurcated graft was then sutured to the E-Vita Open prosthesis. Finally, side anastomosis between the trifurcated graft and aortic graft was completed and CPB was continued via RAA (Figure 1, Step 3).

### 2.3. Definition of Events

Early mortality was defined as death occurring within 30 days or during the admission index. Stroke was defined as the presence of neurological symptoms for more than 24 h. The cause of stroke (ischemic or hemorrhagic) was defined based upon evidence from cerebral computed tomography scans. Technical success was defined as successful aortic debranching and stent-graft placement in descending thoracic aorta.

### 2.4. Statistical Analysis

Patient data were collected using Microsoft Office Excel 2007, version 12. Continuous variables were reported as mean and standard deviation (SD). Categorical variables were reported as numbers and percentages. Due to the small number of patients, there were no sufficient data to perform a more detailed analysis regarding the perioperative outcome.

## 3. Results

The mean age of patients was 61 years ± 6.7 years, and half of patients were males. One patient was referred with malperfusion syndrome and one patient came with recent stroke. Eight patients were referred on emergency basis because of a type A acute aortic dissection (*n* = 4) and PAU (*n* = 4). Demographic data and aortic disease are reported in Table 1.

Four patients had previously undergone cardiac operations. Concomitant cardiac procedures were aortic root replacement in 6 patients (Bentall operation in 4 patients and David operation in 2 patients), ascending aorta replacement in 3 patients, and coronary artery bypass grafting in 3 patients. Table 2 reports the perioperative data.

Early death occurred in 1 patient on 4th postoperative day because of multi-organ failure. No patient underwent re-operation for postoperative bleeding and no patient experienced ischemic or hemorrhagic stroke. Postoperative outcomes are listed in Table 3.

One patient died from a fatal hemorrhagic stroke 24 months after surgery.

## 4. Discussion

In this study we describe the “beating heart debranching first” technique and the clinical outcomes of 12 patients who underwent this procedure.

The debranching of the arch vessels has several advantages including: (1) the risk of cerebral embolisms from aortic debris may be reduced; (2) the myocardium can be perfused during the reimplantation of the supra-aortic vessels, facilitating the management of CPB and myocardial ischemic times; (3) hemostasis of each anastomosis is technically easier [6]; (4) the “beating heart debranching first” technique allows for the continuous perfusion of supra-aortic vessels, optimizing neuroprotection [7].

Open surgery is the “gold standard” for the treatment of arch disease. Surgery requires extracorporeal circulation with hypothermia and circulatory arrest and revascularization of the supra-aortic arteries. There is a need to perform an “elephant trunk” or a “frozen elephant trunk” when the descending aorta is also involved. Due to advances in brain protection techniques, these procedures have achieved good results in high-volume centers with extensive experience. However, overall results in the daily practice are still mixed. In addition, concomitant cardiac disease, advanced age, and comorbidities make the operation more challenging, with an increasing risk of early mortality.

One relevant issue is the cerebral protection technique. Technical improvements in cerebral perfusion and protection have led to important reductions in morbidity and mortality rates for open surgery performed in high-volume centers. There is also evidence that selective anterograde selective perfusion seems to produce better results, especially when the left subclavian artery (LSA) is also selectively perfused.

More recently, some centers have included supra-aortic vessel reimplantation/debranching as part of cerebral protection in order to better manage selective vessel perfusion and to perform the shortest possible ischemic hypothermic arrest. The “frozen elephant trunk” operation seems to be safer and less time-consuming, with better results. It is possible to perform an easier anastomosis in zone 0 or 1 and consequently shorten splanchnic ischemic duration. The International E-Vita Open Registry clinical results observed a permanent spinal cord injury rate reduction from 6% to 3% in two study periods (2005–2011 and 2012–2019) and this reduction was possibly related to a significant reduction in visceral ischemic time (from 66 min to 56 min) [8] with half of the operations performed at 20–25 °C systemic hypothermia [9]. With anastomosis in zone 0 or 1 and retrograde perfusion via the femoral artery, the duration of low body ischemic time can be dramatically reduced. In addition, this approach allows the surgery to be performed at a warmer level of hypothermia, from 28 °C to 34 °C (mild hypothermia), with a reduction in the rate of permanent and temporary postoperative neurological deficit, postoperative dialysis, the duration of ventilation, and intensive care unit stay [10].

It is recommended that the reimplantation of the LSA and selective cerebrospinal be performed, as well as the cerebrospinal fluid (CSF) drainage, which will be used depending on the length of the descending aorta that is covered, whether there is adequate revascularization of the LSA, if the patient has undergone prior abdominal aortic surgery (lumbar arteries occlusion), or if the hypogastric arteries are occluded.

With the “debranching first” technique, continuous perfusion of supra-aortic vessels can be allowed. Cerebral blood flow is never interrupted during the entire procedure. In addition, a no-touch preparation for perfusion of the supra-aortic vessel can be performed, reducing the risk of embolization. The collateral flow of supra-aortic vessels through the cranio-facial collateral network plays an important role in the “debranching first” approach. While right hemispheric perfusion is warranted by direct cannulation of the RAA, the very proximal position of the clamp on the left carotid artery allows the rich collateral networks around the head and neck to increase left hemispheric blood perfusion beyond the perfusion provided by the circle of Willis. Blood from the right carotid and subclavian arteries returns to the left carotid artery via the extracranial vessel network. This context is different from that of carotid endarterectomy, in which the distal common and external carotid arteries are clamped, and an intact circle of Willis is relied on exclusively for collateral supply [3,11]. Finally, because of the continuous anterograde cerebral flow, the degree of hypothermia can be targeted to protect the lower part of the body and the heart, with different clinical advantages. Retrograde perfusion via femoral artery allows for very short ischemic times on lower body regions (only the time needed to insert hybrid or standard prosthesis inside the descending aorta). Perioperative hypotension and prolonged ischemia of the lower half of the body are crucial factors. Both are potentially avoidable, with the prompt recovery of pelvic and lower limb perfusion serving as a measure to reduce the incidence of spinal cord ischemia. The rate of spinal cord ischemia was shown to be reduced from 25% to 2% when this measure was taken along with CSF drainage, an aggressive transfusion protocol, and the maintenance of a mean arterial blood pressure above 85 mmHg. In this evidence, continuous cerebral flow (with LSA always perfused) and a very short lower body ischemic time allow for temperature management that makes the operation feasible and safe, probably even under in cases normothermia and without CSF drainage.

Another issue is myocardial protection in such complex surgery involving the aortic arch and other myocardial or vascular structures. These operations are often extremely time-consuming in terms of CPB and cross-clamping time. On the one hand, with the debranching first technique, it is possible to perform it with a beating heart and thus reduce CPB time; on the other hand, it is possible to address the other “cardiac” diseases in a step “before” the distal arch to allow reperfusion of the heart immediately after completion of the “proximal” step through an adapted cardioplegic line. In this way, it is possible to complete the operation with a relatively short cross-clamping time and reduce the risk of myocardial impairment after surgery.

Over the past decade, interest in endovascular and hybrid approaches for aortic arch repair has grown, especially in patients at high surgical risk. Endovascular repair of the TAAD with the so-called Endo-Bentall was first described by Ryliski et al. [12,13] and consists of a transapical approach to deploying an endovascular valve conduit for the treatment of aortic valve and ascending aorta diseases. Two clinical scenarios for the endo-Bentall approach have been described: (a) to stabilize the ascending aorta in patients without malperfusion syndrome at high surgical risk; (b) as a first-step option to treat distal organ malperfusion by re-expanding the true lumen, temporally stabilizing the ascending aorta and the patient, and postponing conventional surgery to a later date. Endovascular approaches for arch repair with fenestrated and branched stent-grafts is an emerging option for high-risk patients. However, these approaches are not currently widely used due to the lack of “universality” of the manufacture of device and the need to create a custom-made device that takes time to be produced by industries. In addition, the lack of large follow-ups investigating the sealing of the graft on the wall of the ascending aorta is currently a limitation to the wider application of these devices [14].

An endovascular approach for arch repair with fenestrated and branched stent-grafts is an emerging option for high-risk patients. However, these approaches are not currently widely used due to the lack of “universality” of the manufacture of device and the need to create a custom-made device that takes time to be produced by industries. In addition, the lack of large follow-ups investigating the sealing of the graft on the wall of the ascending aorta are currently a limitation to the wider application of these devices [14].

This report presents some limitations. Firstly, this is a retrospective monocentric analysis and therefore there is an inherent risk of bias due to the nature of the study. Secondly, the small number of patients and the unmeasured variables do not allow for detailed statistical analysis. Thirdly, this study reports an initial experience; therefore, results may be affected by a natural learning curve. However, the peri- and postoperative outcomes are consistent with results reported from high-volume centers. Lastly, the lack of a long-term follow-up does not allow us to identify such patients who received long-term benefit from this technique.

## 5. Conclusions

“Debranching first” techniques for supra-aortic vessels in beating hearts and during cardiopulmonary bypasses are feasible operation for managing complex diseases involving the aortic arch. It reduces the need for hypothermia and decreases both cardiopulmonary bypass and myocardial arrest times. We believe this approach could represent an affordable surgical option, even in high-risk patients, for the treatment of aortic arch disease. However, longer follow-ups and further reports from other centers adopting this technique are encouraged.

## Figures and Tables

**Table 1:** Demographic variables.

Variables	Patients (*n* = 12)
Age (years), mean (SD)	61 (6.7)
Male (%)	6 (50)
LVEF, mean (SD)	56.6 (6.8)
Obesity (%)	1 (8.3)
Hypertension (%)	7 (66)
CAD (%)	3 (25)
Malperfusion (%)	1 (8.3)
Stroke (%)	1 (8.3)
Malperfusion syndrome (%)	2 (8.3)
Euroscore II, mean (SD)	10.1 (7.4)
**Aortic disease**	
PAU	4 (33.3)
Type A acute aortic dissection (%)	4 (33.3)
Chronic aortic dissection	2 (16.6)
Aortic aneurysm with arch involvement	2 (16.6)

LVEF, left ventricle ejection fraction; CAD, coronary artery disease; PAU, penetrating aortic ulcer; SD, standard deviation.

**Table 2:** Perioperative data.

Variables	Patients (*n* = 12)
Emergency (%)	8 (66.6)
Reintervention (%)	4 (33.3)
Bentall (%)	4 (33.3)
David (%)	3 (25%)
CABG (%)	3 (25)
Ascending Aorta replacement (%)	3 (25
Operation time, minutes (SD)	467 (33)
CPB time, minutes (SD)	206 (30.7)
Aortic-Clamp time, minutes (SD)	93 (41.7)
CA time, minutes (SD)	14.3 (9.6)
Nasopharyngeal Temperature, °C (SD)	34 (0.6)

CABG, coronary artery bypass grafting; CPB, cardiopulmonary bypass time; SD, standard deviation; CA, circulatory arrest.

**Table 3:** Postoperative data.

Variables	Patients (*n* = 12)
Early mortality (%)	1 (8.3)
Stroke (%)	0
TND (%)	1 (8.3)
Reoperation for postoperative bleeding (%)	0
AKI (%)	0
Ventilation time, days (SD)	2.8 (2.1)
Transfusion with RBC (%)	11 (91.6)
ICU stay, days (SD)	7.5 (4.2)
Hospital stay, days (SD)	13.7 (7.5)

TND, transient neurological defect; AKI, acute kidney injury; ICU, intensive care unit; RBC, red blood cell; SD, standard deviation.

## Data Availability

The data presented in this study are available on request from the corresponding author.
